# Fatal VAP-related pulmonary aspergillosis by *Aspergillus niger* in a positive COVID-19 patient

**DOI:** 10.1016/j.rmcr.2021.101367

**Published:** 2021-02-18

**Authors:** Laura Trovato, Maddalena Calvo, Giuseppe Migliorisi, Marinella Astuto, Francesco Oliveri, Salvatore Oliveri

**Affiliations:** a U.O.C. Laboratory Analysis Unit, A.O.U. “Policlinico-Vittorio Emanuele”, Via S. Sofia 78, Catania, 95123, Italy; b Department of Biomedical and Biotechnological Sciences, University of Catania, Via S. Sofia 97, Catania, 95123, Italy; c Department of Anesthesia and Intensive Care, A.O.U. “Policlinico –Vittorio Emanuele”, Via S. Sofia 78, Catania, 95123, Italy

**Keywords:** SARS-CoV-2, Coinfection, Ventilator-associated pneumonia, Pulmonary aspergillosis, *Aspergillus niger*, IPA, Invasive pulmonary aspergillosis, ICU, Intensive Care Unit, C-PAP, Continuous Positive Airway Pressure, GM, *Aspergillus* antigen galactomannan, VAP, ventilator associated pneumonia

## Abstract

Invasive pulmonary aspergillosis, known as a complication in patients with severe respiratory syndromes, recently showed a correlation with COVID-19 pneumonia, and the clinical characteristics of COVID-19 associated pulmonary aspergillosis (CAPA) have been described. Unfortunately, infections by the *Aspergillus* genus are often diagnosed in *post-mortem* time, because of diagnostic delays and a rapid worsening of respiratory conditions. Literature data document, in fact, only few cases of COVID-19 *Aspergillus niger* coinfection. The aim of this study was to describe a case of a VAP-related probable pulmonary aspergillosis by *Aspergillus niger* in a COVID-19 patient. Despite the definition of fungal etiology and the rapid administration of antifungal therapy, the patient died while on ventilator support because of severe respiratory impairment.

## 1 Background

IPA, caused by the *Aspergillus* genus, is known as a common complication in patients with severe respiratory syndromes and is also related to high mortality rates [1,2]. There are many predisposing factors to the development of IPA, basically recognized in prolonged treatment with corticosteroids and lung epithelial damage [1]. Several cases of IPA have been documented as super-infections in patients with severe respiratory illness such as influenza and MERS-CoV [1,3]. Starting in December 2019 many severe respiratory syndrome cases caused by Coronavirus-19 (SARS-CoV-2) have been diagnosed. The clinical impact of this infection defines a highly dysregulated immune response and diffuse lung damage, which lead to the early onset of secondary infections [2,3]. Here we describe a case of invasive pulmonary aspergillosis by *Aspergillus niger* in a patient with COVID-19 pneumonia and acute respiratory distress syndrome.

## 2 Case presentation

**Fig. 1:**
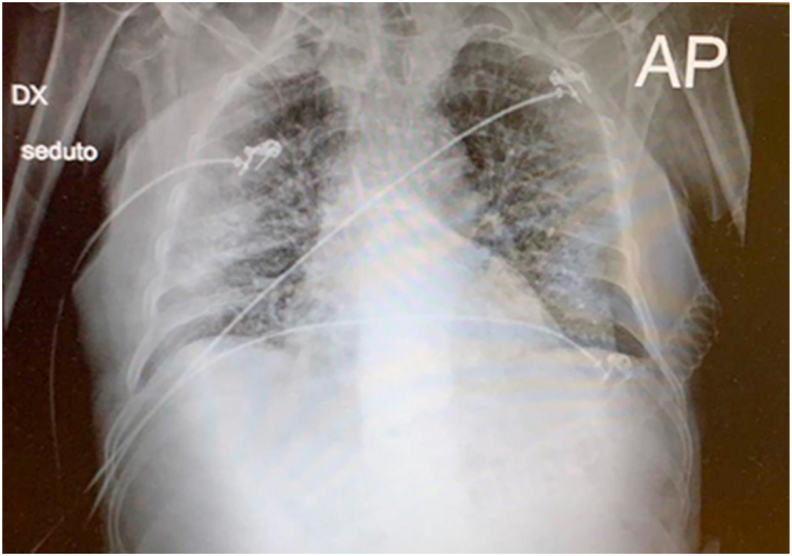
Rx-graphic performed in anteroposterior projection and in seated decubitus with a portable device. A parenchymal consolidation is visible in the right-middle lung field. There is also an extended interstitial lung disease with a reticulo-nodular pattern, pronounced pulmonary hilar and free costo-phrenic angles. Cardiac image is enlarged.

In October 2020, a 73-year-old man was admitted to the accident and emergency department of the University Hospital of Catania, Sicily, Italy, reporting fever, cough and diarrhea. Vital signs were recorded as the following: blood pressure of 160/87 mmHg, heart rate of 85 beats per minute, respiratory rate of 40 beats per minute and SPo2 of 78%. A chest X-ray showed bilateral infiltrates (Fig. 1) and a nasopharyngeal swab sample was collected and tested positive for COVID-19 using molecular testing.

**Fig. 2:**
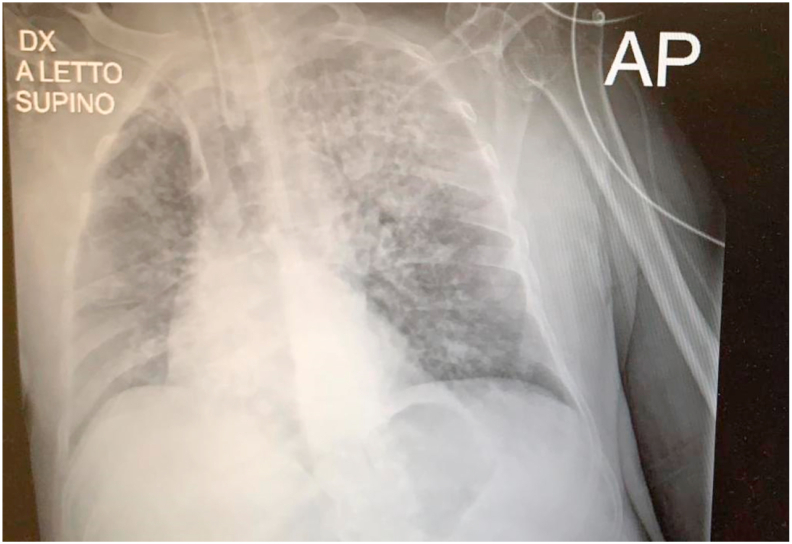
Rx-graphic performed in anteroposterior projection and in supine decubitus with a portable device. An extended parenchymal consolidation is visible in left apical, intercleidohilar and hilum-para-hilar point. A shadowed parenchymal consolidation is also visible in the right intercleidohilar point. There is an extended interstitial lung disease with a reticulo-nodular pattern. Because of cardiac image overlapping, the right hilum cannot be evaluated. Left hilum is large and thickened. Right costo-phrenic angles are partially hidden.

The patient was moved to the ICU with the diagnosis of Coronavirus-19 pneumonia. Clinical history was updated to include a previous diagnosis of diabetes and hypertension. Corticosteroid therapy with dexamethasone (12 mg/day) and C-PAP ventilation were immediately required. In the following days, laboratory tests showed significant increases of white blood cells (up to 30210/mm3) and lactate (up to 864 U/L), and low albumin (down to 2.27 g/dL). There was also a high increase of C-reactive protein up to 101.87 mg/L. On day 4, seric levels of the GM (Platelia *Aspergillus*; Biorad) and 1,3-β-D-glucan (Fungitell; Associates of Cape Cod Inc., Falmouth, Massachusetts, USA) were prescribed because of the patient's risk factors, which tested negative. On day 10 ventilator support with oro-tracheal intubation was implemented, due to a rapid decline of the patient's consciousness and respiratory quality. Because of a fever episode, antibiotic treatment with meropenem (3g/day) was started and a peripheral blood sample was taken for a microbiological culture, which tested negative. On the same day some surveillance exams were performed: cultures from rectal, nasal and pharyngeal swabs reported a normal microbiota, while a culture from a urinary sample revealed 100000 ufc/ml of *Pseudomonas aeruginosa*. Because of this positive result the urinary catheter was removed and a therapeutic lavage with antibiotics was carried out, with complete resolution. On day 16 a new chest X-ray was performed that showed a pulmonary worsening (Fig. 2).

**Fig. 3:**
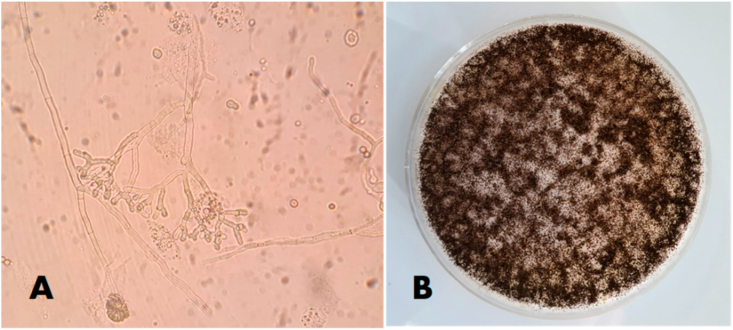
(A) Direct examination of bronchoaspirate sample with 15% KOH (original magnification ×40); (B) growth on Sabouraud Dextrose Agar after 72 h at 32 °C.

At the same time, new seric dosages were required: β-glucan serum level was 84 pg/mL while galactomannan had a T index of 4.9, suggestive of probable fungal angioinvasion. A bronchoaspirate sample was collected for bacteriological and mycological examinations, because of the persistence of high inflammation indices. For the mycological examination conventional methods by microscopic and fungal culture in Sabouraud's dextrose agar medium supplemented with chloramphenicol and gentamycin were performed. Molecular methods were also used and real-time PCR assay for the detection of *Aspergillus* was carried out. The AsperGenius® multiplex PCR (PathoNostics, The Netherlands) was used for the detection of the most clinically relevant *Aspergillus* species. DNA was extracted by using the GenoXtract instrument (Hain Lifescience, Germany) following the manufacturer's instructions. PCR was performed adding 5 μl of DNA extract to the PCR mix and a Rotor-Gene Q (Qiagen) was used for amplification and melting curve analysis. The direct microscopic examination with 15% potassium hydroxide (KOH) showed several hyaline septate hyphae and *Aspergillus* sp. was detected by PCR. After 72 h of incubation at 32 °C, the growth of numerous colonies was observed (Fig. 3).

Identification of the isolate was performed by standard phenotypic methods, based on macroscopic and microscopic morphological studies. The pathogen was identified as *Aspergillus* section *Nigri*. Matrix-assisted laser desorption ionization time of flight mass spectrometry on a Microflex LT (Bruker Daltonics, Bremen, Germany) platform after ethanol-formic acid extraction, identified the isolate as *Aspergillus niger* (score: 2.155). The same sample reported negative results from bacteriological examinations. Surveillance cultures from oropharyngeal, rectal and nasal swabs also revealed the presence of *Aspergillus niger*. Susceptibility to fluconazole, itraconazole, voriconazole, posaconazole, flucytosine, caspofungin, anidulafungin, micafungin, and amphotericin B, was evaluated by the Sensititre®YeastOne method. MIC values of >256 μg/mL for fluconazole, 0.25 μg/mL for itraconazole, 1 μg/mL for voriconazole, 0.125 μg/mL for posaconazole, 2 μg/mL for flucytosine, and 0.12 μg/mL for amphotericin B were obtained. Echinocandins presented adequate minimum effective concentration (MEC) values: 0.03 μg/mL for micafungin, 0.015 μg/mL for caspofungin and 0.03 μg/mL for anidulafungin. Results showed various therapeutic options evaluated with dilution ranges and epidemiological cut-offs in the absence of clinical breakpoints [4]. Some literature reports evaluated the effectiveness of isavuconazole against the *Aspergillus* genus and the reliability of the MIC strip method for its susceptibility test [5,6]. According to these reports, the susceptibility test for isavuconazole was performed using the MIC strip method and showed a MIC value of 2 mg/L. The patient was put on voriconazole 800 mg/day. On day 19, the patient died in the ICU of heart failure, while he was still on ventilator support.

## 3 Discussion and conclusion

According to recent literature data (June 2020), about 38 cases of COVID-19 associated pulmonary aspergillosis are known [1,2]. Percentages are probably underestimated owing to a diagnostic delay or to the lack of clinical recognition [1]. Most COVID-related pulmonary aspergillosis cases are, in fact, belatedly diagnosed, often in post-mortem time. In our case, because of the patient's critical issues, it was not possible to collect samples from the lower respiratory tract regularly. This inconvenience had a negative impact on the possibility to define a fungal colonization by *Aspergillus* and on the timeliness of a correct diagnosis of pulmonary aspergillosis. These delays, together with patient's risk factors represented by extensive lung damage and prolonged treatment with corticosteroids [3], involved a rapid worsening of the respiratory condition. The patient also reported a previous diagnosis of diabetes, which is related to structural modifications of blood vessels and predisposes to fungal angioinvasion. To clarify the eventuality of a previous chronic *Aspergillus* colonization, a detection of *Aspergillus*-specific antibodies by agar gel immunodiffusion was performed on serum. The negative result leads us to the assumption that the patient, most likely, was infected in a hospital setting during intubation. According to recent data *Aspergillus* sp. are recognized as a potential cause of VAP in immunocompetent hosts [7,8]. Unfortunately, fungi are often not included among the possible causes of VAP in non-immunocompromised patients with other risk factors and therefore *Aspergillus* shows its angioinvasive properties for a long period before the real diagnosis of pulmonary aspergillosis [8]. Assuming that the infection of our patient was a VAP-related pulmonary aspergillosis, a frequent check of seric and colonization parameters would have allowed a prompter diagnosis: in the six days between oro-tracheal intubation and diagnosis of pulmonary aspergillosis, we do not have regular reporting of a mycological surveillance for this patient. This omission led to a dangerous consequence: *Aspergillus* had sufficient time to proliferate and to angioinvade the patient's respiratory tract, therefore the diagnosis and the administration of voriconazole were not enough, also considering the critical status of the pulmonary epithelium. Notwithstanding his critical clinical condition, the patient contrasted the progression of the infection for a brief time, both because of the absence of a marked neutropenia, which is often a predisposing condition to pulmonary aspergillosis and because of the involvement of *Aspergillus niger*, whose virulent nature is widely confirmed but it is lower when compared to other *Aspergillus* species. In fact, although *Aspergillus niger* is able to produce a severe pulmonary disease it is rarely reported as a cause of invasive aspergillosis, while it is often described as the etiological agent of otomycosis and cutaneous infections [9]. Moreover, in a study that investigated the presence of triazole-resistant *Aspergillus* isolates in agricultural areas in Southern Italy, the presence of other *Aspergillus* species was reported. These species, although less pathogenic than *A. fumigatus*, are very frequently isolated in Sicily and can represent a potential cause of invasive disease in patients at risk [10]. Despite the definition of fungal etiology and the rapid administration of voriconazole, the patient died because of the severe impairment of his respiratory condition. Consequently, invasive pulmonary aspergillosis should be investigated as a possible complication in cases of severe respiratory syndromes, even in immunocompetent hosts [11,12]. A rapid diagnosis can lead to the development of an accurate therapeutic plan and probably to clinical remission only if the patient has no comorbidities nor extensive pulmonary damage. Through our experience we suggest adding a study of fungal colonization to the clinical management of all immunocompetent patients with one or more risk factors for the development of invasive fungal infection [13]. Immunocompetent patients with COVID-19 infection should be screened early for microbiological colonization before admission to critical wards, such as ICUs. Colonization data allow a carefully monitoring of the patient and prevention of invasive infections, especially in cases on ventilator support.

## Funding

This research received no external funding.

## Author contributions

Investigation, Methodology and Writing the original draft of the manuscript: L.T., M.C., and G.M.; Data curation: F.O. and M.A.; Methodology and Supervision: S.O. All authors read and approved the final manuscript.

## Declaration of competing interest

The authors declare that they have no known competing financial interests or personal relationships that could have appeared to influence the work reported in this paper.
